# Stakeholder perceptions of preventive approaches to rheumatoid arthritis: qualitative study of healthcare professionals’ perspectives on predictive and preventive strategies

**DOI:** 10.1186/s41927-023-00361-8

**Published:** 2023-10-03

**Authors:** Imogen Wells, Gwenda Simons, Jasin Philip Kanacherril, Christian D. Mallen, Karim Raza, Marie Falahee

**Affiliations:** 1 Rheumatology Research Group, Institute of Inflammation and Ageing, College of Medical and Dental Sciences, University of Birmingham, Birmingham, B15 2WB UK; 2 Warwick Medical School, University of Warwick, Coventry, UK; 3 Birmingham Medical School, University of Birmingham, Birmingham, UK; 4 School of Medicine, Keele University, Keele, UK; 5 NIHR Birmingham Biomedical Research Centre, University Hospitals Birmingham NHS Foundation Trust and University of Birmingham, Birmingham, UK; 6 Sandwell and West Birmingham NHS Trust, Birmingham, UK; 7 MRC Versus Arthritis Centre for Musculoskeletal Ageing Research and the Research into Inflammatory Arthritis Centre Versus Arthritis, University of Birmingham, Birmingham, UK

**Keywords:** Rheumatoid arthritis, Interview, Qualitative, Predictive tools, Preventive treatment, Healthcare professionals

## Abstract

**Background:**

There is increasing research interest in the development of preventive treatment for individuals at risk of rheumatoid arthritis (RA). Previous studies have explored the perceptions of at-risk groups and patients about predictive and preventive strategies for RA, but little is known about health care professionals’ (HCPs) perspectives.

**Methods:**

One-to-one semi-structured qualitative interviews were conducted (face-to-face or by telephone) with HCPs. Audio recordings of the interviews were transcribed, and the data were analysed by thematic analysis.

**Results:**

Nineteen HCPs (11 female) were interviewed, including ten GPs, six rheumatologists and three rheumatology nurse specialists. The thematic analysis identified four organising themes: 1) Attributes of predictive and preventive approaches; 2) Ethical and psychological concerns; 3) Implementation issues and 4) Learning from management of other conditions. Theme 1 described necessary attributes of predictive and preventive approaches, including the type and performance of predictive tools, the need for a sound evidence base and consideration of risks and benefits associated with preventive treatment. Theme 2 described the ethical and psycho-social concerns that interviewees raised, including the potential negative economic, financial and psychological effects of risk disclosure for ‘at-risk’ individuals, uncertainty around the development of RA and the potential for benefit associated with the treatments being considered. Theme 3 describes the implementation issues considered, including knowledge and training needs, costs and resource implications of implementing predictive and preventive approaches, the role of different types of HCPs, guidelines and tools needed, and patient characteristics relating to the appropriateness of preventive treatments. Theme 4 describes lessons that could be learned from interviewees’ experiences of prediction and prevention in other disease areas, including how preventive treatment is prescribed, existing guidelines and tools for other diseases and issues relating to risk communication.

**Conclusions:**

For successful implementation of predictive and preventative approaches in RA, HCPs need appropriate training about use and interpretation of predictive tools, communication of results to at-risk individuals, and options for intervention. Evidence of cost-efficiency, appropriate resource allocation, adaptation of official guidelines and careful consideration of the at-risk individuals’ psycho-social needs are also needed.

**Supplementary Information:**

The online version contains supplementary material available at 10.1186/s41927-023-00361-8.

## Background

Rheumatoid arthritis (RA) is a chronic inflammatory joint disease, which causes joint pain, swelling, stiffness and fatigue, as well as joint damage [1, 2] and extra-articular features such as cardiovascular and pulmonary disease which may reduce life expectancy [3, 4].

Advances in understanding of the biological mechanisms underlying the development of RA [5–7] and prediction of who is likely to develop RA in the future [8–11] have laid the foundations for increased research focus on interventions for at-risk individuals to prevent or delay disease development and progression [12, 13]. A number of clinical trials of pharmaceutical preventive treatments are either completed or underway to investigate this, including in asymptomatic first-degree relatives [14]. Early findings are promising and may signify a shift towards RA prediction and prevention, rather than treatment of established RA in future years.

At present, the responsibilities of healthcare professionals (HCPs) focus primarily on the diagnosis and management of RA, including identification of signs and symptoms of RA, communication of treatment options, and provision and monitoring of pharmacological treatments [15]. HCPs may also recommend lifestyle changes such as smoking cessation and weight loss that support the effective management of RA. As the introduction of predictive and preventive approaches would likely affect HCPs’ roles and responsibilities in both primary and secondary care, it is important to understand their views around such approaches for RA. Understanding these views will help to identify potential barriers and facilitators, and support needs that would need to be addressed to inform the design and implementation of effective approaches.

A small number of studies have explored the perceptions and preferences towards predictive and preventive strategies for RA among several different stakeholders. These stakeholders include members of the public, at-risk groups such as first-degree relatives (FDRs) of patients with RA and those with clinically suspect arthralgia (CSA), and RA patients. Stakeholders identified several concerns relating to the accuracy and certainty of the risk information provided by predictive tools, as well as the potential for these tools to cause psychological harm to a person or their family [16–19]. Compared to at-risk groups who were asymptomatic (such as FDRs), those with symptoms were more likely to take a predictive tool or preventive treatment for RA [18]. Stakeholders also reported a preference for lifestyle compared to pharmacological interventions to reduce the risk of RA [20, 21]. The efficacy of preventive treatments in reducing RA risk was identified as important in stakeholder’s decision-making surrounding the uptake of these treatments [20, 21].

A very small number of studies have included assessment of rheumatologists’ perceptions about predictive and preventive approaches for RA in addition to FDRs and RA patients. Rheumatologists highlighted concerns regarding the cost and accuracy of predictive tools as well as a lack of evidence surrounding the efficacy and safety of pharmacological treatments to reduce risk [22–24]. Similar to other stakeholders, this group also expressed a preference for lifestyle interventions [23, 24]. However, differences in preferences between rheumatologists, FDRs and RA patients were also identified; rheumatologists placed more emphasis on the certainty of evidence surrounding preventive treatment compared to FDRs and RA patients, whereas FDRs and RA patients placed greater importance on how a treatment is taken [23]. Rheumatologists were also more likely to choose a preventive treatment for at-risk individuals over no treatment compared to FDRs and RA patients [23]. It is therefore important that the perspectives of all stakeholders are explored and understood.

To our knowledge, no study to date has examined the perspectives of other relevant HCPs, including those from primary care services, towards predictive and preventive approaches to RA. Consequently, the aims of the present study were to explore the perceptions of rheumatologists, specialist nurses and GPs regarding the utility of predictive and preventive approaches for RA, and factors that may affect their implementation within healthcare services.

## Methods

### Design

This study was a qualitative interview study using a semi-structured interview schedule [25]. Data were analysed thematically using the approach developed by Braun and Clarke, [26] with codes and themes identified using an inductive approach, based on the data obtained in this study [26–28].

Ethical approval was granted by the University of Birmingham Science, Technology, Engineering and Mathematics Ethical Review Committee (ERN_18-1781). The Consolidated Criteria for Reporting Qualitative Research (COREQ) guidelines [29] were used to report on the methods and results of this study.

### Participant recruitment

HCPs were eligible to take part in the interviews if they: (1) managed patients with RA within primary or secondary care settings, and (2) were proficient enough in English to participate in an interview. HCPs who work predominantly with patients under the age of 18 were not eligible to participate.

A sample size of around 10–20 interviews has been suggested as sufficient to achieve data saturation for this type of study [30, 31]. In the current study, interviews were conducted until data saturation was achieved, determined by consensus among the research team that no new information was generated in the interviews, and no new codes or themes were identified in the analysis [32].

GPs were identified either through the NIHR CRN (West Midlands), or with support from CM (an NIHR professor of general practice) at the Midlands Partnership NHS Foundation Trust (MPFT), who identified individuals who met the inclusion criteria for the study and contacted them via email on behalf of the research team. Rheumatologists and rheumatology nurse specialists were identified by KR (a senior consultant in rheumatology) at Sandwell and West Birmingham (SWB) NHS Trust, and by research staff at MPFT, who contacted potential participants either face-to-face or via email.

Participants were recruited using a convenience sampling technique, [33] and were made aware that the interviewer was a PhD student supervised by KR (a senior rheumatology consultant) and MF (a research psychologist specialising in rheumatology).

### Procedure

Participants were provided with a background questionnaire and consent form to complete prior to their interview. The background questionnaire assessed gender, professional role, years since qualification and whether the participant had a specialist interest in rheumatology. All interviews were conducted by IW, a female PhD student with a background in health psychology and experience in conducting one-to-one interviews. The interviews were conducted either face-to-face at a mutually agreed location or over the phone and only IW and the participant were present during the interviews. No repeat interviews were carried out. Field notes were made by IW after each interview.

The interviews were guided by a semi-structured interview schedule. The initial draft of the interview schedule was developed by IW, with input from MF and KR. Several open-ended questions were developed to address the study aims, with the first half of the schedule focused on predictive approaches for RA, and the second half addressing preventive treatment. A small number of vignettes relating to the provision of predictive strategies for those at various stages of RA risk (those who presented with RA symptoms, and those who presented with a family history of RA only) were also included.

The questions generated were informed by previous related studies [16, 17, 34]. and through involvement of a panel of stakeholders including an FDR of an RA patient, a GP, a rheumatology nurse specialist, and a rheumatologist, none of whom were participants in the study. As a result of the input of the stakeholders, the interview schedule was updated to [1] clarify the type of preventive interventions the research team wished to discuss, including pharmaceutical treatments, lifestyle interventions, or both, and [2] include prompts relating to the type of predictive tool that HCPs may consider (for example inflammatory markers or imaging). In addition, a face-to-face pilot interview with a rheumatology nurse specialist was also conducted to determine the effectiveness of the interview schedule at addressing the aims of the study. Following feedback from this interview, the schedule was modified further to include interview prompts to further clarify questions relating to perceived challenges and benefits associated with preventive interventions. The final interview schedule is provided in supplementary materials 1.

All interviews were audio-recorded and transcribed verbatim using an independent transcription company (The Transcription Company). Transcripts were not returned to participants for comment and/or correction but were sense checked by the interviewer.

### Analysis

The analysis was facilitated by the NVivo software (version 12.0), which enabled coders to record codes identified from the raw data and arrange them into categories and overarching themes [20]. Three researchers (IW, JPK (a medical student) and GS (a health psychologist)) read the transcripts in full to familiarise themselves with the data, and then coded the data line by line. There was an overlap of three of the 19 transcripts which were independently coded by IW, JK and GS without a codebook to assess consistency. The independent coding was compared for the selected transcripts and discussed amongst the three coders to identify inconsistencies. It was agreed that all codes were comparable in meaning, and any differences related only to the description of the codes. A common terminology was agreed and reviewed by a fourth researcher (MF). This was then used to guide further coding.

The resulting codes, and the field notes made after each interview were used to develop initial themes and subthemes, which were continuously refined and developed through regular discussions with KR and MF. This was facilitated by a document collating coded data extracts from all interviews organised into overarching categories. The themes were then further refined by GS using this collated document. IW reviewed these additional refinements, and the final themes were decided upon through discussions with GS, IW and MF. Participants did not provide feedback on the findings. Data analysis was conducted in parallel with data collection to facilitate assessment and revision of the semi-structured interview schedule, if necessary, as well as to assess whether data saturation had been achieved.

## Results

### Participants

Nineteen HCPs (11 female) from the Midlands (UK) took part in a one-to-one interview either over the phone (*n* = 18) or in person (*n* = 1) between November 2019 and July 2021. No new codes or themes were identified from transcripts of the last three interviews conducted, which was interpreted as evidence of data saturation. Participants included ten GPs, six rheumatologists and three rheumatology nurse specialists with an average of 14 years of experience in healthcare post qualification at the time of the interviews. Interviews lasted between 30 and 80 min. Although most interviews were conducted prior to the onset of the COVID pandemic, three interviews with rheumatologists were conducted during the pandemic in July 2021. The characteristics of individual HCPs are summarised in Table 1.

**Table 1:** Participant characteristics

Participant ID number	Gender	Role	Years since qualification
1	Male	Rheumatologist	8
2	Female	Rheumatology clinical nurse specialist	23
3	Male	Rheumatologist	
4	Female	Rheumatology clinical nurse specialist	12
5	Female	Rheumatology clinical nurse specialist	16
6	Female	GP	9
7	Male	GP	10
8	Female	Rheumatologist	20
9	Male	GP	7
10	Female	GP	0.8
11	Female	GP	10
12	Female	GP	9
13	Male	GP	29
14	Male	GP	20
15	Female	GP	14
16	Female	GP	6
17	Female	Rheumatologist	21
18	Male	Rheumatologist	24
19	Male	Rheumatologist	11

‘−‘ indicates missing data

### Thematic data analysis

The analyses resulted in four organising themes, each with a number of subthemes (Table 2). The first theme, ‘Attributes of predictive and preventive approaches’, encompasses HCPs’ views on those aspects of predictive tools, preventive treatments and other interventions that were considered important. Theme two, ‘Ethical and psychosocial concerns’, deals with HCPs’ views about the potential psychological and social/ ethical consequences of predictive approaches. Theme three, ‘Implementation issues’, covers what HCPs described as necessary to integrate predictive and preventive approaches into RA care and the impact it might have on healthcare services. Finally, theme four, ‘Learning from management of other conditions’, describes the lessons that can be learned from HCPs’ experiences of predictive and preventive approaches in other disease areas.

**Table 2:** Overview of organising themes and subthemes

Organising themes	Themes
1) Attributes of predictive and preventive approaches	1. Type and performance characteristics of predictive tool 2. Availability of and evidence base for preventive approaches 3. Risks associated with treatment 4. Benefits of predictive tools and preventive treatments
2) Ethical and psychological concerns	1. Social consequences 2. Psychological consequences 3. Uncertainty
3) Implementation issues	1. Knowledge of prediction and prevention and training needs 2. Resources 3. HCP roles 4. Guidelines 5. Patient factors
4) Learning from management of other conditions	1. Knowledge of disease 2. Treatment 3. Guidelines and tools for other diseases 4. Risk communication

### 1) Attributes of predictive and preventive approaches

This theme deals with the attributes of predictive and preventive approaches the interviewees considered important. The four subthemes are outlined below and supporting quotes can be found in the text and in Table 3.

**Table 3:** Quotes illustrating Theme 1 ‘Attributes of predictive and preventive approaches’

PPN	Quote
1	“Those would be blood tests and rheumatoid factor, particularly CCP to look for an inflammatory response”, PPN 1, Rheumatologist.
2	“Sometimes it can be quite difficult to interpret because I don’t think we fully understand ultrasound findings for example. But I think that often we get inflammatory ultrasound findings in osteoarthritis. And you know non rheumatoid, and I don’t think we’re particularly good at really being able to distinguish in some cases, you know what is significant of what I’m finding and what isn’t. […] inflammatory markers, you know they’re not, I’m sure you know, they’re not particularly discriminatory and also people who are overweight, they’re often elevated so, if they’re slightly raised, it can be quite hard I think to inform decision making using, you know the results are equivocal.” PPN 17, Rheumatologist.
3	“So, I think the predictive tests, to be useful and beneficial, have to be sufficiently strong […] in their conclusions that they are useful in either making a negative or positive decision say to treat someone who is going to develop a rapidly progressing disease early to make a lifestyle change early” PPN 01, Rheumatologist.
4	“Even if you’ve got predictive tests, I don’t think you could predict how severe, you know, each individual patient was going to be, just put them in high risk you don’t know if they might develop a more aggressive disease. I don’t think you can really predict onset of how bad it can be you know. It’s tricky.” PPN 05, Rheumatology clinical nurse specialist.
5	“I probably wouldn’t, given our current provision of services, refer somebody who had a marker that said they were likely to develop something unless there was a new strategy that meant that we could act on that.” PPN 11, GP
6	“So, we would want some level of assurance that actually using these drugs in these patients is going to lead some benefit and what are the mitigations for those who never actually develop rheumatoid arthritis? We’ve exposed them to these toxic medications when they didn’t necessarily require them.” PPN 19, Rheumatologist.
7	“If they haven’t got any signs or symptoms alongside the risk, we wouldn’t want to give medications at this stage because these are life-long medications that can have an effect on the blood and body.” PPN 04, Rheumatology clinical nurse specialist.
8	“Your risk of giving somebody a preventive intervention for a disease they haven’t got and they develop a side effect is not great for the patient or the clinician.” PPN 08, Rheumatologist.
9	“You know the other side of this, the disadvantages as well as the anxiety and all the rest of it, it’s a kind of it’s people describe in research terms of treatment burden. But it’s actually, having to attend appointments, having to, you know take medicine, having to have blood tests, having all that time and commitment and work, that’s the other kind of downside of things, isn’t it, that?” PPN 17, Rheumatologist.
10	“I mean there might be positive changes about it might help them to adopt more positive lifestyle behaviours. Like smoking, something about dental health as well because I know that you know people with poor oral dental health have a higher risk of getting RA, particularly if they’ve got anti-CCP so I don’t know if there’s something preventative there around best dental health, I don’t know.” PPN17, Rheumatologist.
11	“It means the patient isn’t exposed to the pain, disability and longer-term consequences of what is a very unpleasant, chronic condition.” PPN 06, GP
12	“It’s not just about the direct illness. It’s almost the other things that happen with RA. People get cardiovascular disease; they get lung disease; they get heart problems. It’s not just about the specific disease itself which would improve outcomes much more long-term for people.” PPN 07, GP
13	“I do wonder if you had somebody that you knew was at risk of development something, then they would know what to look out for and they would be aware that they would need to seek help and advice, perhaps, at the early signs.” PPN 10, GP

**Type and performance characteristics of predictive tool.** HCPs, and in particular rheumatologists, discussed a number of factors they deemed important for predictive tools. They considered factors such as: What type of predictive tools might be appropriate, describing blood tests including autoantibodies (Table 3, Quote 1; T3Q1); Potential issues with interpretation of test results, for example an elevation in inflammatory markers not being specific for inflammatory arthritis (T3Q2); And the performance characteristics of available tools themselves including the need for appropriately high positive and negative predictive values (T3Q3). Complicating matters for many interviewees, was a recognition that RA itself was a heterogenous disease and that tests may not be able to predict who might be likely to face a severe disease course versus those who would be likely to face a mild course (T3Q4).

**Availability of, and evidence base for, preventive approaches.** Several HCPs stated that risk assessments should only be carried out if preventive interventions are available (T3Q5). Others mentioned that they would need to be assured that such intervention would have sufficient benefit and were worried about exposing individuals who might not actually require treatment to unnecessary side effects from drug treatments (T3Q6). Interviewees also discussed the need for evidence of the preventive treatment’s effectiveness, and concerns about how long the preventive treatment may need to be given for:

*“I think for preventive interventions, you probably wouldn’t want to treat somebody for longer than a year or two years without clear evidence to do that”.* PPN 08, Rheumatologist.

**Risks associated with the preventive treatment.** GPs, rheumatologists, and nurse specialists all recognised that preventive treatments (some of which currently under consideration are treatments for established RA) come with risks of (potentially severe) side effects:

*“So, the DMARDs, are, you know, particularly unpleasant drugs and I regularly see patients in practice who experience significant side effects from them and end up having two, three before they, you know, get put onto something, you know, immunological agents*.” PPN06, GP.

Some suggested they would not expose patients to such treatments if they did not have symptoms (T3Q7-8). Some also recognised the burden that the preventive treatment might pose for at risk individuals, including having to take the medicine, time commitment and having to be monitored (T3Q9), which might prevent treatment uptake.

**Perceived benefits of predictive tools and preventive treatments**. HCPs also described potential positive outcomes of predictive testing and preventive treatments. Some HCPs hoped that risk assessment would influence people’s lifestyle choices and lead to changes in behaviour such as smoking cessation (T3Q10) which in turn might reduce their chances of developing RA. Preventing future pain and suffering for patients was also a priority for interviewees (T3Q11). HCPs also recognised that by reducing the risk of RA you also reduce the risk of co-morbidities of RA such as cardiovascular disease (T3Q12). Another positive consideration, identified in particular by GPs, was that if patients knew about their risk, they would be more likely to be vigilant for symptoms and present early if they developed them (T3Q13). For many the possibility of earlier intervention if RA were to develop, and potential prevention of RA, were seen as the main benefit of predictive tools.

### 2) Ethical and psychosocial concerns

This theme describes potential psychological and social/ ethical consequences of predictive approaches. The three subthemes are outlined below and supporting quotes can be found in Table 4.

**Table 4:** Theme 2: Ethical and psychological concerns

PPN	Quote
1	“I would also say that there are not maybe now but, in the future, there will be insurance implications of knowing potentially that you have a risk of developing a disease like rheumatoid which is associated with morbidity and increased health costs and an insurance company would definitely be very keen to know that information”. PPN 01, Rheumatologist
2	“They could say, ‘We know that you’re going to get RA and therefore, we’re not going to insure you, sorry. We’re not going to give you health insurance.‘ There are some unintended consequences from that point of view because actually having a diagnosis or a pre-diagnosis may have implications on all that sort of thing.” PPN 07, GP.
3	“There are other issues around things like mortgage applications, life insurance, jobs, that are they going to have to declare something that they don’t have currently, but they may be at risk of developing, which will make their life more difficult, make it harder to get loans, make it harder for them to buy a house or, you know, work in the area they want to work in as well.” PPN 09, GP.
4	“In asymptomatic people, I guess it can create a certain amount of anxiety and concern for the patient”. PPN 03, Rheumatology clinical nurse specialist
5	“It causes a lot of anxiety and stress for these patients as well, to be told that they could have positive RA.” PPN 15, GP
6	“We’ve just got to remember that these are predictive risks. I would imagine there will be some sort of threshold but it’s not going to be saying they will get rheumatoid arthritis.” PPN 07, GP
7	“So, I suppose the main issue is that they are predictive. They’re not guaranteed. So, we’re saying to someone, ‘You have a risk of so much of developing this condition in the future, but we can’t guarantee that you actually are going to get it’.” PPN 19, Rheumatologist.
8	“Even if you’ve got predictive tests, I don’t think you could predict how severe, you know, each individual patient was going to be just put them in high risk you don’t know if they might develop a more aggressive disease. I don’t think you can really predict onset of how bad it can be you know.” It’s tricky. PPN 05, Rheumatology clinical nurse specialist
9	“So, my understanding of those tests, particularly the blood tests, they’re not very specific.” PPN 16, GP.
10	“You can get positive rheumatoid factors, and nothing comes of it. Are we then putting patients at risk of going on treatments and having steroids? It wouldn’t have been needed if we’d have just waited.” PPN 04, Rheumatology clinical nurse specialist

**Social/ ethical consequences.** Interviewees discussed the social consequences of predictive tools and subsequent treatment including the potential implications for health or life insurance availability or costs (T4Q1-2) or potential impact on future employment prospects (T4Q3). Some participants expressed concerns that the results of predictive tools could raise problematic questions, for example if it were possible to determine someone’s future risk of RA at a (very) early age, should we offer treatment at that very early point:

*“Let’s say you develop a predictive test, but it’s actually genetic based and you could even look at the foetus to determine their risk of rheumatoid arthritis in due course. If you could say that they are at risk of developing severe rheumatoid arthritis from their early 20s onwards, there’s then very serious questions about, well, do we offer treatment from birth, or do we even allow the birth to proceed? There’s potentially quite severe knock-on consequences for these sorts of tests.”* PPN 18, Rheumatologist

**Psychological consequences**. Interviewees discussed the potential for negative psychological consequences of risk communication, (T4Q4-5), especially in relation to uncertainty around disease development:

*“I really wouldn’t want to do a test that would give them a nebulous risk of you know developing a condition that they might never get* […] *and that would cause anxiety and aside from the lifestyle changes I’d recommend anyway”.* PPN 01, Rheumatologist.

**Uncertainty.** HCPs described concerns about the uncertainty associated with risk information, particularly in relation to the inability to guarantee that RA would or would not develop (T4Q6-7). They also described additional uncertainty around issues such as the likely severity of RA if it were to develop (T4Q8), and lack of specificity of predictive tools that are currently available (T4Q9). Such considerations were associated with concerns around the potential for overtreatment of individuals identified as being at risk to prevent RA development (T4Q10).

### 3) Implementation issues

This theme centres around what would be needed for effective integration of predictive and preventative approaches to RA, and the potential impact of these approaches on the health service. The five subthemes are outlined below and supporting quotes can be found in Tables 5 and 6.

**Table 5:** Theme 3: Implementation issues (Knowledge and training needs; HCP roles)

PPN	Quote
1	“I’m not particularly aware of any risk prediction tools for patients who are still asymptomatic” PPN 06, GP
2	“I know we are looking into it and blood tests can predict which type of RA you’ve got which driver you have you know.” PPN 05, Rheumatology clinical nurse specialist.
3	“You can do a combination of tests and anti CCP antibodies obviously increases your risk of developing RA in the future and there are a variety of different HLA proteins but again, these aren’t widely used in clinical practice for the prediction of RA.” PPN 08, Rheumatologist
4	“I don’t really think we’ve got any tools at the moment really. Certainly not in clinical practice really, you know we have no screen tools, I guess if you know that somebody has particular risk factors, you know whether that’s, I guess gender, is it a sort of, or biological sectors as sort of, weak, well it’s not weak, in the sense of rheumatoid is more common in women than men, you know smoking, I guess there are certain things, but we really don’t have any screening tools in clinical practice at the moment.” PPN 18, Rheumatologist.
5	“I don’t know what you can do in terms of preventing RA but as far as I know there’s not much you can do.” PPN 12, GP
6	“Not very much, I would say. I mean I guess that I guess there might be lifestyle interventions, so for example in somebody who is high risk for example, or somebody’s got a strong family history, you know might be advisable for them to stop smoking, I suppose. In terms of drug treatments, I don’t know an awful lot, I think there have been some trials, but it’s not something I’ve looked at in any detail.” PPN 18, Rheumatologist.
7	“I know there have been studies done looking at giving intramuscular steroids for patients with symptoms and I mean positive serology but not actual joint inflammation. I know there is the APPIPRA study, but I don’t think the results are there yet.” PPN 03, Rheumatologist.
8	“The evidence base isn’t strong. I think there is some evidence that it [methotrexate] delays the time to onset but not that is changes the eventual outcome.” PPN 08, Rheumatologist.
9	“And so, it’s [counselling around treatment] very un-patient centred, and it doesn’t allow patients to discuss concerns. And it’s also heavily weighted on harm of drugs rather than benefits. So, that approach really wouldn’t work for this preventative medicine, because you’ve really, as I’ve said before, got this nuance of the patient and what the potential benefit is, again, you know which is going to be hard to get across, because it will be about not necessarily immediate gain but future gain, balance against the burden of side effects of medicine. So, I think it’s a very, doesn’t have to be a medic, but it’s a very skilled conversation.” PPN 17, Rheumatologist.
10	“I think general nurses might need a little bit more input you know, with those communication skills, the ability to handle this kind of information [risk information]” PPN 05, Rheumatology clinical nurse specialist.
11	“I think it’s just down to having those skills to manage that situation, knowing it might upset the patient and the patient being in denial […] so maybe a bit more training how to do that” PPN 05, Rheumatology clinical nurse specialist
12	“The only thing I would say in primary care that I would be willing to offer is like a lifestyle intervention, you know, going through risks and being able to say that you know, stop smoking, lose weight, they should be general lifestyle changes anyway that we recommend to everyone.” PPN 15, GP
13	“This [lifestyle intervention] is something we do all the time, every day and would do it sort of routinely with patients so smoking cessation is something that, yeah, it’s bread and butter general practice.” PPN 06, GP.
14	“Proper counselling is often better done by specialist nurses really than doctors. Doctors can be a bit blunt about these things sometimes.” PPN 07, GP
15	“I think nurse appointments. I don’t know for sure, but I believe are a bit longer and I hope that… they certainly seem to do in this trust and some others, they actually talk about those things [lifestyle interventions] and they talk about the importance of activity and things.” PPN 01, Rheumatologist.
16	With exercise, like I say, the physios should have an input. I think when we’re looking at that, perhaps more patients should have a chance to see a physio and get advice from that point of view and perhaps see the occupational therapist at the same time. PPN 04, Rheumatology clinical nurse specialist.
17	“Also, what the implications of that might be. So, and then equally, that healthcare professional needs to have the, you know, sort of skills and the knowledge to interpret the test correctly and then know what to do but I guess, you know I don’t think that necessarily needs to be a doctor, doesn’t necessarily need to be a rheumatologist, you can sort of see that other members of the team, nurses in particular, you know might be possible, they might be able to do this.” PPN 18, Rheumatologist.
18	“I would advise them [patient] that the GP would be the first point of call for assessment, and they will give you tests and if any of that was positive then the GP would refer them on.” PPN 05, Rheumatology clinical nurse specialist.
19	“I think it [a predictive test] would determine how quickly I would refer them. So obviously if they were positive and indicative of rheumatoid arthritis, I’d be more likely to refer them urgently. But it sounds like they need a rheumatology referral anyway.” PPN 16, GP

**Table 6:** Theme 3: Implementation issues (Resources; Guidelines; Patient factors)

PPN	Quote
1	“There are costs to the patient in terms of monitoring requirements and costs to the health service in terms of monitoring requirements, like chest x-rays and that sort of thing.” PPN 08, Rheumatologist
2	“Clearly, we’d be using very high-cost drugs thinking about rituximab for a much bigger proportion of the population and depending on the threshold at which you set your criteria for access to those drugs.” PPN 11, GP
3	“If you can demonstrate a marginal benefit people will say well that’s better than nothing […] and then you can persuade funders or insurance companies to allow that treatment to be used”, PPN 01, Rheumatologist
4	“The funding aspect, so getting CCG to pay for drugs for practice on diseases that they’ve not yet got might be a challenge”. PPN 05, Rheumatology clinical nurse specialist
5	“Ideally, in a properly funded service, you’d like to be proactive and the identifying people before they’ve developed something that’s going to cause them major problems, rather than waiting for them to get it.” PPN 09, GP
6	“You need to know quite a lot of detail about what the test is going to be able to do and how beneficial their treatment was in terms of cost benefit in reducing the need for services.” PPN 06, GP
7	“It would need to integrated properly into the system, you need pay the professionals properly to do it and you need to give them time to do it, you can’t just add it on with everything else, into the GPs contract without any recognition of extra workload. PPN 12, GP
8	“Do you need more clinic space? Do you need more secretarial support? Do you need more specialists in certain types of lab testing?” PPN 10, GP
9	“There are costs to patients in terms of monitoring requirements and costs to the health service in terms of monitoring requirements, like chest x-rays or that sort of thing.” PPN 08, Rheumatologist.
10	“Unless there was a very, very strong indication and very, very low risk of using these medications and a very clear, agreed pathway for CCG, whoever it will be at that time, with how it’s prescribed and given. PPN 06, GP
11	“I think in terms of existing services, I think the treatment could, you know streamlined and integrated efficiently within existing pathways for treatments that departments already have.” PPN 18, Rheumatologist
12	“I think predictive testing does have an important role, but I think it needs to be taken up and integrated into our national guidelines like NICE etc.” PPN 12, GP
13	“They then need to be integrated into recognised guidelines if you want them to be taken up by practitioners I think.” PPN 12, GP.
14	“[If] In their family we have a high risk of developing the disease, it’s important to get those patients seen and tested earlier rather than waiting for symptom onset or you know, the disease to establish.“ PPN 05, Rheumatology clinical nurse specialist.
15	“You’d be less inclined to give the treatment to a patient that’s 20 years old and is not going to get rheumatoid arthritis until they’re 80. You’d wait until they’re 79 to give them preventive treatment, wouldn’t you? PPN 03, Rheumatologist
16	“So, I think explaining risk, communicating risk is particularly difficult ‘cause I think you have to have quite a high level of health literacy and numerical literacy. I would take it on a patient-by-patient approach as to how I explain risk, especially when you’re getting into sensitivities and specificities of testing.” PPN 06, GP.

**Knowledge of prediction and prevention and training needs.** A number of HCPs indicated that they were not aware of existing risk prediction tools for asymptomatic individuals (T5Q1), whereas others mentioned using the currently available diagnostic tools for RA (T5Q2). Some rheumatologists described existing predictive tools but often noting that these are not integrated into routine care (T5Q3-4).

Whereas some HCPs described a lack of awareness of preventive strategies for RA (T5Q5), other than the possibility for lifestyle interventions (T5Q6), several rheumatologists in the current study were aware of clinical trials looking at RA prevention (T5Q7). Some were also aware that the evidence base for preventive interventions is very limited at present (T5Q8).

HCPs further identified specific training needs to be met for them to deliver predictive and preventive approaches for RA effectively. These include training in communication of risks and benefits associated with preventive interventions (T5Q9-10) and providing psychological support to patients (T5Q11). HCPs also stated they would need training on how to use predictive tools, and interpret the results:

“*We would need training and education in how to use the tests and what the results actually mean, and then we would require capacity to do that from a clinic setting.”* PPN 19, Rheumatologist.

**HCP roles.** HCPs identified potential responsibilities they could take on in the prediction and prevention of RA, as well as those for other HCPs. GPs felt they were well placed to prescribe lifestyle interventions, (T5Q12-13) pharmacological interventions were perceived to be more appropriately provided in secondary care:

*“If it’s drugs, then I would say that currently, unless the methotrexate and rituximab come with very, very, very specific instructions, then I’d still suspect that that would need to be done in secondary care, or certainly initiated in secondary care”* (PPN 09, GP).

Specialist nurses were seen as particularly well suited to have discussions with patients around risk information and preventive treatment for RA, as they may be likely to spend more time with patients than other HCPs and have the relevant skills to discuss this information in a sensitive manner (T5Q14-15). Interviewees also identified other HCPs such as occupational therapists and physiotherapists who could play a role in facilitating lifestyle interventions to prevent RA development (T5Q16). HCPs felt that tests could be interpreted accurately by a range of HCPs as long as they received appropriate training (T5Q17). HCPs also described how they would refer a patient/ an individual at high risk of RA to a rheumatologist (T5Q18-19).

**Resources.** This subtheme incorporates discussion of the potential costs (T6Q1-2) and funding implications (T6Q3-5) of the integration of predictive and preventive measures into the UK health care systems. HCPs invariably felt that the measures need to be cost effective and that there needed to be robust evidence of cost effectiveness (T6Q6). However, the potential for significant benefit for patients and the wider society was highlighted:

“*Because in terms of all risk, if it can be treated sooner or treatment before it kicks in, it’s obviously better for the patient and the healthcare economy in the long term.”* PPN 05, Rheumatology clinical nurse specialist.

HCPs further discussed that appropriate resources would need to be allocated to support the increased demands that would be needed to support effective implementation of preventive strategies, such as staff time and expertise, workload, clinic space, and administrative support (T6Q7-8). They also highlighted that requirement for monitoring patients at risk of developing RA would be associated with costs both for the health service and patients themselves (T6Q9). Some HCPs further suggested there might be a need for dedicated RA prevention clinics:

“*So, again, as mentioned before, whether we need to then run dedicated clinics or whether we can swap this in amongst our early arthritis or general clinics. I suspect, given that it’s a completely different way of thinking for us, it might be helpful to have its own dedicated clinic, in which case do we need a trained consultant who only deals with that, or a trained nurse who only deals with that?”* PPN 18, Rheumatologist.

**Guidelines.** The need for a clear pathway for management of predictive and preventive interventions alongside existing pathways for RA was discussed (T6Q10-11). To effectively integrate these approaches into the healthcare system, it was noted that they would need to be addressed in extended national treatment guidelines (T6Q12-13).

**Patient factors influencing predictive approaches and preventive treatment prescription.** HCPs also described patient factors that could affect both HCP and patient decision-making about predictive tools or preventive treatment. These included family history of RA (T6Q14), age (T6Q15) and treatment preferences of the individual at risk:

*“I think if we can prevent it, it would be good as long as the patient is happy to take those medications to prevent it.”* PPN 04, Rheumatology clinical nurse specialist.

HCPs further shared ideas on how the results of a predictive tool, or risk associated with a treatment plan would need to be communicated to the at-risk individual in a way that would suit the individual and was tailored to their level of understanding/ education and experience (T6Q16).

### 4) Learning from the management of other conditions

The final overarching theme centres around what HCPs felt could be learned from their experiences of predictive and preventive approaches in other disease areas. The four subthemes are outlined below with supporting quotes in Table 7.

**Table 7:** Theme 4: Lessons learned from other disease areas

PPN	Quote
1	“I guess if you compare it to the diabetes literature where you identify people before they’ve got diabetes and they’ve determined pre-diabetes and we’re starting to treat people now with pre-diabetes. So, I guess if the science is similar, maybe you get to a stage where you have pre-RA”. PPN 16, GP.
2	“I think, generally speaking, patients know about the risk of developing diabetes, heart disease, and things like that and can buy into preventive actions for that. I think RA is poorly understood at a population level and so I think patients would struggle to appreciate where RA fits in.” PPN08, rheumatologist.
3	“With something like a cardiac event, if you’ve got a 10% cardiac risk, over a ten-year period this is, then we should be giving people statins which they have to take on a daily basis but actually, most people don’t even notice it. Actually, the biggest faff about it is taking it on a daily basis and remembering because there are no consequences to that. It’s that kind of balance and it really depends on the toxicity of the treatment to prevent RA.” PPN 07, GP.
4	“Hopefully it [lifestyle interventions for RA] would be something like pre-diabetes where you’ve identified that risk, you go on a diabetes prevention course and that’s enough for the patients to change their behaviour so that they don’t become diabetic, that’s what I’d say from a prevention course” PPN 15, GP.
5	“So, it would be entirely something that the patient should be consulted with at every step of this, rather than it being something that we are doing. My feeling is it’s like statins; the requirement of the guidance was about the risk of a heart attack was cut from 20–10% over ten years and it effectively meant that, you know, every single male over 60, regardless of how healthy they were, should be on a statin. At which point, I don’t think that’s individualised or personalised medicine, I think that’s just pathologising old age, and so I think that if it was done in a personalised way that the individual had a proper understating of exactly what their risk was and exactly what the benefit was, then that’s a situation that […] with these medications.” PPN 09, GP.
6	“For example, a two-week wait is the famous one where people come in with altered bowel habits. I think the risk of actually having a cancer with altered bowel habits is about 5%. All of a sudden, you’re having to do 20 colonoscopies or CT scans to pick that one cancer up. It’s going to massively increase it [use of RA tests]”. PPN 07, GP.
7	“So, take a population of people similar to the person in front of me and estimate it over a period of time, cardiovascular disease for example ten years and show how many of that group would then turn out to have the condition and then if there was an intervention how many of those people would be helped. So cardiovascular disease, 10% risk over ten years you’d have 100 people, 10 would look glum at the end of a 10-year period.” PPN 13, GP.
8	“When we’re talking about the risk of stroke with the DOACs and stuff like that, we’re talking about a 4% or 5% risk. When you see the smiley faces and the sad faces, you might be getting four sad faces of getting a stroke. You’re given the medication and now two people are having the stroke. On 100 faces, it doesn’t look like an awful lot, but you could say, ‘This is a 50% reduction of your risk,‘ or something like that.” PPN 07, GP.

**Knowledge of disease.** HCPs referred to their knowledge of research into preventive strategies and experience of the clinical translation of that research in other disease areas (T7Q1). HCPs stated that members of the public tended to be less knowledgeable about RA compared with other diseases such as diabetes mellitus (DM) and cardiovascular disease (CVD) and highlighted the impact that this lack of knowledge may have on the uptake of preventive approaches (T7Q2). It was further suggested that the lower prevalence of RA compared to other disease areas limited the amount of evidence for preventive strategies:

*“We’ve been able to research that* [CVD] *quite robustly with the tools we’ve got because it’s such a common disease. Whereas, with rheumatoid arthritis, it’s quite rare actually, isn’t it?”* PPN 07, GP.

**Treatment.** HCPs described their experiences of prescribing preventive interventions for other (chronic) diseases, for example statins to reduce risk of CVD and identified similarities in how they would approach the issue of risk-benefit trade-offs in the context of decisions about preventive treatment for RA (T7Q3). Some favoured lifestyle interventions (T7Q4) or regular monitoring of patients identified as being at risk over preventive pharmacological interventions, describing applications of such approaches in practice or research studies for other conditions, such as diabetes:

*“I’m aware of a study being done in type 1 diabetes where they look for a genetic marker and if that genetic marker is present then they do a blood test every year to see if antibodies start to be developed. So, they’re doing sort of blood testing as opposed to exposing patients to treatment, so I see that as being perhaps a better and less burdensome way of managing potential risk.”* PPN 06, GP.

**Guidelines and tools for other diseases.** HCPs discussed their experience of working with existing guidelines for the prediction and prevention of other diseases such as bowel cancer and CVD and used that experience to make suggestions for guidelines for the prediction and prevention of RA as well as pointing out the potential pitfalls of such guidelines. HCPs highlighted the importance of personalised approaches and effective risk/benefit communication to facilitate shared decision making but also warned of over-medicalising healthy individuals, by putting them on preventive treatment (T7Q5). HCPs further worried about burdening the health system, highlighting guidelines that suggested the need to further screen individuals with what they described as non-specific symptoms. (T7Q6)

Existing tools that assess risk were also discussed as examples of predictive tools already integrated into clinical practice, in particular the widespread use of the QRISK score for classifying those at risk of developing CVD:

*How are they* [HCPs] *going to identify it* [RA risk] *so is it a clinical scoring tool, in cardiovascular, you use QRISK, is there a clinical scoring risk tool for that…?* PPN 15, GP.

**Risk communication.** The final lesson learned from other disease areas was about how risk information and risk reduction strategies could be communicated to those at risk of RA most effectively. HCPs made suggestions based on their experiences of communicating risk for chronic diseases such as CVD and osteoporosis (T7Q7-8), for example, using ‘smiley/sad face’ pictograms or descriptions:

*“Well, I haven’t done this within the context of inflammatory arthritis. I mean I do a lot about communication in osteoporosis. … I think there’s lots of sort of generic challenges, even though I don’t know much about the, you know the early inflammatory arthritis example. I mean the first thing is explaining what the condition is that you’re trying to predict and what the significance of that is. And then depending on how the risk is, then you’d be trying to explain that in an understandable way as possible, not using percentages or anything but talking, you know the numeric risks using some simple frequencies.”* PPN 17, Rheumatologist.

## Discussion

The findings from the current study increase our understanding of the views of HCPs who would likely be involved in the prescription of predictive and preventive approaches for RA. The interview data show that in order to successfully implement predictive and preventive approaches for RA in the current UK healthcare system a number of factors need to be considered. HCPs had clear views about the necessary attributes of predictive and preventive approaches, including the sensitivity of the predictive tool and the need for a robust evidence base for the preventive approach as well as consideration of both risks and benefits associated with preventive treatment. They further raised ethical and psycho-social concerns that they felt needed to be taken into consideration, including the potential negative effects of risk disclosure for the individual, existing uncertainty around the risk of developing RA, the potential for harm (side-effects) and the potential for benefit associated with the treatments being considered. The interviews also revealed a number of implementation issues, including the need for appropriate resource allocation, guidelines, and training around predictive tools and treatment, including interpretation and communication of risk results to patients. Interviewees’ responses were informed by experiences of preventive approaches in other disease areas.

The concerns regarding the accuracy and certainty of RA risk information provided by predictive tools identified in the current study are consistent with results from previous studies examining the perceptions of rheumatologists, members of the public, RA patients and their relatives [16–19, 22–24]. The need to develop tools that provide high positive and high negative predictive values are important to ensure the success of these approaches. However, given the heterogenous nature of RA, this may be difficult to achieve [35]. The need to establish the cost-effectiveness of preventive approaches for RA was also consistent with previous research, where cost-effectiveness was identified as an important factor in decision-making around preventive treatment for RA among RA patients and those at risk [20, 21].

Similarly, HCPs’ concerns around the potential for predictive tool results to cause psychological harm to patients align with previous findings from studies examining perceptions of members of the public, RA patients and their relatives [16–19]. Appropriate support should therefore be provided to the at-risk individuals alongside risk communication. This could be provided by HCPs involved in communicating risk information to patients though they would need to receive appropriate training and tools.

Many HCPs in the current study preferred the idea of prescribing lifestyle-related treatment or regular monitoring for those at risk of RA (to allow early intervention when RA developed) rather than pharmacological preventive interventions. In contrast, participants in studies around preventive treatments for CVD expressed a preference for pharmacological treatment compared to lifestyle-based interventions [36].

### Strengths and limitations

Using qualitative interviews combined with an inductive thematic analytical approach provided the opportunity for new concepts to be explored in depth with a different group of stakeholders [27, 31], generating rich and informative data. This study further benefits from extensive research partner involvement in the design of the interview schedule. Furthermore, the results from the study represent the perceptions of HCPs with varying degrees of experiences and a variety of relevant healthcare roles.

However, there are some limitations. Firstly, all interviews were conducted with HCPs who worked in a healthcare setting within the Midlands, UK. Their views may not be representative of HCPs working in other regions. Further research is needed to understand the views of healthcare professionals in other regions within the UK and in other nations with different healthcare systems. Further studies are also needed to understand the views of other types of HCPs involved in management of RA who were not represented here, such as physiotherapists and occupational therapists. Secondly, the use of a convenience sample in this study could have led to potential bias in the types of participants recruited. As such, their views and motivations may not reflect all relevant HCPs. Furthermore, it is possible that some participants were more exposed to research in the area of interest than is typical, as they were recruited through clinical members of research team. However, this is less likely to be the case amongst GP participants, who comprise over half of the sample and are less likely to be familiar with the research area. Thirdly, the predominant use of telephone interviews within this study may have impacted on the data received due to the lack of non-verbal cues and rapport generally gained through face-to-face contact. However, telephone interviews can still provide rich, detailed and high-quality data [37]. Finally, we did not collect data on how many RA patients were in contact with the HCPs in our study in a typical week, or the type of hospital/primary care setting that participants worked in. Further studies are needed to explore how these, and other contextual variables are associated with perceptual variation.

## Conclusions

To ensure the successful implementation of predictive and preventative approaches for RA, HCPs across primary and secondary care services need appropriate training around predictive tools, interpretation of results, communication of results to at-risk individuals, and options for preventive interventions. There is a further need for evidence of cost-efficiency of preventive approaches. Appropriate resource allocation and development of national guidelines are also needed, along with the development of risk communication tools and psychosocial support resources. In designing preventive services for RA, much can be learned from other chronic disease areas such as CVD. Implementation studies that take into account the needs identified by HCPs in the current study are required to inform the development of effective future strategies that will be widely accepted and applied within healthcare services.

### Electronic supplementary material

Below is the link to the electronic supplementary material.

Supplementary Material 1

## Data Availability

The data underlying this article will be shared on reasonable request to the corresponding author.

## Declarations

All methods were carried out in accordance with relevant guidelines and regulations.

## Abbreviations

Anti-CCP: Anti Cyclic Citrullinated Peptide
CCG: Clinical Commissioning Groups
COREQ: Consolidated Criteria for Reporting Qualitative Research
CVD: Cardiovascular disease
DMARD: Disease Modifying Anti Rheumatic Drug
DM: Diabetes Mellitus
FDR: First Degree Relative
GP: General Practitioners
HCP: Health Care Professional
NICE: National Institute for Health and Care Excellence
NIHR CRN: National Institute for Health and Care Research Clinical Research Network
PPI: Patient and Public Involvement
MPFT: Midlands Partnership Foundation Trust
RA: Rheumatoid Arthritis
SWB: Sandwell West Birmingham

## References

[1] Grassi W, De Angelis R, Lamanna G, Cervini C (1998). The clinical features of rheumatoid arthritis. Eur J Radiol.

[2] Schett G, Gravallese E (2012). Bone erosion in rheumatoid arthritis: mechanisms, diagnosis and treatment. Nat Rev Rheumatol.

[3] John H, Toms TE, Kitas GD (2011). Rheumatoid arthritis: is it a coronary heart disease equivalent?. Curr Opin Cardiol.

[4] Bendstrup E, Møller J, Kronborg-White S, Prior TS, Hyldgaard C. Interstitial lung disease in rheumatoid arthritis remains a challenge for clinicians. J Clin Med. 2019;8(12). 10.3390/jcm8122038 PMC6947091 31766446

[5] van der Woude D, van der Helm-van Mil AHM (2018). Update on the epidemiology, risk factors, and disease outcomes of rheumatoid arthritis. Best Pract Res Clin Rheumatol.

[6] Viatte S, Plant D, Han B, Fu B, Yarwood A, Thomson W (2015). Association of HLA-DRB1 haplotypes with rheumatoid arthritis severity, mortality, and treatment response. JAMA.

[7] Kurowska W, Kuca-Warnawin EH, Radzikowska A, Maśliński W (2017). The role of anti-citrullinated protein antibodies (ACPA) in the pathogenesis of rheumatoid arthritis. Cent Eur J Immunol.

[8] National Insitute for Health and Care Research. PREVeNT RA 2021 [Available from: https://nottinghambrc.nihr.ac.uk/take-part/live-trials/trials/1429-prevent-ra.

[9] Arthritis Check-up. Arthritis-Checkup: Study of an early detection of the disease [Available from: http://www.arthritis-checkup.ch/index_gb.html.].

[10] Kolfenbach JR, Deane KD, Derber LA, O’Donnell C, Weisman MH, Buckner JH (2009). A prospective approach to investigating the natural history of preclinical rheumatoid arthritis (RA) using first-degree relatives of probands with RA. Arthritis Rheum.

[11] van Boheemen L, van Schaardenburg D (2019). Predicting Rheumatoid Arthritis in At-risk individuals. Clin Ther.

[12] Van Steenbergen HW, Da Silva JAP, Huizinga TW, van der Helm-van Mil AH (2018). Preventing progression from arthralgia to arthritis: targeting the right patients. Nat Rev Rheumatol.

[13] Stanway JA, Isaacs JD (2020). Tolerance-inducing medicines in autoimmunity: rheumatology and beyond. Lancet Rheumatol.

[14] National Insitute of Allergy and Infectious Diseases. Strategy to Prevent the Onset of Clinically-Apparent Rheumatoid Arthritis (StopRA) [Available from: https://clinicaltrials.gov/ct2/show/NCT02603146].

[15] Smolen JS, Landewé RBM, Bijlsma JWJ, Burmester GR, Dougados M, Kerschbaumer A (2020). EULAR recommendations for the management of rheumatoid arthritis with synthetic and biological disease-modifying antirheumatic drugs: 2019 update. Ann Rheum Dis.

[16] Stack RJ, Stoffer M, Englbrecht M, Mosor E, Falahee M, Simons G (2016). Perceptions of risk and predictive testing held by the first-degree relatives of patients with rheumatoid arthritis in England, Austria and Germany: a qualitative study. BMJ Open.

[17] Falahee M, Simons G, Buckley CD, Hansson M, Stack RJ, Raza K (2017). Patients’ perceptions of their relatives’ risk of developing rheumatoid arthritis and of the potential for risk communication, prediction, and Modulation. Arthritis Care Res (Hoboken).

[18] Mosor E, Stoffer-Marx M, Steiner G, Raza K, Stack RJ, Simons G (2020). I would never take preventive medication! Perspectives and information needs of people who underwent predictive tests for rheumatoid arthritis. Arthritis Care Res (Hoboken).

[19] Wells I, Zemedikun DT, Simons G, Stack RJ, Mallen CD, Raza K (2022). Predictors of interest in predictive testing for rheumatoid arthritis among first degree relatives of rheumatoid arthritis patients. Rheumatology (Oxford).

[20] Singhal J, Wells I, Simons G, Wöhlke S, Raza K, Falahee M (2022). Public perceptions of predictive testing for rheumatoid arthritis compared to breast cancer and early-onset Alzheimer’s disease: a qualitative study. BMC Rheumatol.

[21] Simons G, Stack RJ, Stoffer-Marx M, Englbrecht M, Mosor E, Buckley CD (2018). Perceptions of first-degree relatives of patients with rheumatoid arthritis about lifestyle modifications and pharmacological interventions to reduce the risk of rheumatoid arthritis development: a qualitative interview study. BMC Rheumatol.

[22] Munro S, Spooner L, Milbers K, Hudson M, Koehn C, Harrison M (2018). Perspectives of patients, first-degree relatives and rheumatologists on preventive treatments for rheumatoid arthritis: a qualitative analysis. BMC Rheumatol.

[23] Harrison M, Bansback N, Aguiar M, Koehn C, Shojania K, Finckh A (2020). Preferences for treatments to prevent rheumatoid arthritis in Canada and the influence of shared decision-making. Clin Rheumatol.

[24] van Boheemen L, Bolt JW, ter Wee MM, de Jong HM, van de Sande MG, van Schaardenburg D (2020). Patients’ and rheumatologists’ perceptions on preventive intervention in rheumatoid arthritis and axial spondyloarthritis. Arthritis Res Therapy.

[25] Hsiao B, Downs J, Lanyon M, Blalock SJ, Curtis JR, Harrold LR (2022). Rheumatologist and patient Mental Models for treatment of rheumatoid arthritis help explain low treat-to-target rates. ACR Open Rheumatol.

[26] Willig C. EBOOK: introducing qualitative research in psychology. McGraw-hill education (UK); 2013.

[27] Braun V, Clarke V (2006). Using thematic analysis in psychology. Qualitative Res Psychol.

[28] Thomas DR (2006). A general inductive approach for analyzing qualitative evaluation data. Am J Evaluation.

[29] Tong A, Sainsbury P, Craig J (2007). Consolidated criteria for reporting qualitative research (COREQ): a 32-item checklist for interviews and focus groups. Int J Qual Health Care.

[30] Guest G, Bunce A, Johnson L (2006). How many interviews are enough? An experiment with data saturation and variability. Field Methods.

[31] Clarke V, Braun V. Planning and designing qualitative research. Successful qualitative research: a practical guide for beginners. SAGE; 2013. pp. 45–50.

[32] Saunders B, Sim J, Kingstone T, Baker S, Waterfield J, Bartlam B (2018). Saturation in qualitative research: exploring its conceptualization and operationalization. Qual Quant.

[33] Robinson OC (2014). Sampling in interview-based qualitative research: a theoretical and practical guide. Qualitative Res Psychol.

[34] Falahee M, Simons G, Raza K, Stack RJ (2018). Healthcare professionals’ perceptions of risk in the context of genetic testing for the prediction of chronic disease: a qualitative metasynthesis. J Risk Res.

[35] Karlson EW, van Schaardenburg D, van der Helm-van Mil AH (2016). Strategies to predict rheumatoid arthritis development in at-risk populations. Rheumatology (Oxford).

[36] Ju I, Banks E, Calabria B, Ju A, Agostino J, Korda RJ (2018). General practitioners’ perspectives on the prevention of cardiovascular disease: systematic review and thematic synthesis of qualitative studies. BMJ open.

[37] Novick G (2008). Is there a bias against telephone interviews in qualitative research?. Res Nurs Health.

