# Supplementary material for: Stakeholder perceptions of preventive approaches to rheumatoid arthritis: qualitative study of healthcare professionals’ perspectives on predictive and preventive strategies
Source: BMC Rheumatol. 2023 Oct 3;7:35. doi: 10.1186/s41927-023-00361-8 (PMC10548722; doi:10.1186/s41927-023-00361-8)
Supplement: Supplementary file 1 — Supplementary Material 1 [file 41927_2023_361_MOESM1_ESM.docx]

**Supplemental Material 1:** Interview schedule

**Interview Schedule**

**Predictive testing**

**General introduction:**

This study will explore your perceptions towards predictive and preventive interventions for rheumatoid arthritis (RA). There are currently a number of studies working on creating predictive strategies to identify those who are at risk of developing RA in the future. There is also a strong research focus on identifying effective treatments for the early stages of RA, including even before the onset of symptoms in those “at risk” of RA.

In light of these current developments, we would like to gain an understanding of your thoughts about the use of these approaches and their potential impact within clinical practice. This information is important as it can help to inform the design of future predictive and preventive strategies.

The initial questions will relate to your perceptions regarding predictive testing for RA for anyone who might be at risk of RA but hasn’t yet developed it. In this context, predictive testing will include any test that can provide information about whether a person is likely to develop a specific condition in the future.

- **Do you think it’s important to be able to predict RA development in those at risk?**
  - Why/why not?
- **Do you think the tools we have at the moment are adequate**?
  - Why/why not?
  - E.g. bloods for inflammatory markers, ACPA, RF, ultrasound/ MRI scans.
- **What do you currently know about measures that could increase the ability to predict the likelihood of developing Rheumatoid Arthritis?**

You will now be provided with some short scenarios describing individuals who come to you sharing some concerns about their health. Once you have been presented with a scenario, you will be asked a series of questions related to that scenario.

**Vignette 1:**

A patient has mentioned that they find it difficult to get out of bed in the morning because their joints are very stiff. They state that their finger and wrist joints hurt in particular and hurt most when they wake in the morning, but can last all day. The patient has not reported any swelling of the joints and there was none to find on examination.

**Vignette 2:**

A patient comes to you mentioning that they are concerned about developing RA because their mother has been living with RA for a number of years.

**Questions to be asked after the presentation of each vignette:**

1. **What would you do?**

- Prompt: Are there any (other) tests that might be useful in this situation e.g. blood tests (RF, ACPA, inflammatory markers (e.g. CRP/ESR?) or imaging (e.g. ultrasound /MRI)
- Why / why not?

1. **How useful would you find the results of these tests? How would these results impact on your decision making (what you would do next)?**

- Prompt: how useful would a test be which indicates a high likelihood/ low likelihood/ intermediate likelihood that an individual will develop RA?
- What would count as a high/low/intermediate likelihood?

You will now be asked some more general questions about predictive testing:

1. **How likely to develop RA in the future would someone need to be in order for medical action (preventive intervention) to be needed. What action (if any) would be appropriate?**

- For example, would a person need to be 20%, 50%, or 70% likely to develop RA for a preventive intervention to be needed, in your opinion?
- There are no right or wrong answers.

1. **How would you explain the results of predictive tests to patients?**

- Risk score, graphical, other?

1. **How may measures that increase the current ability to predict that someone will develop RA in the future affect healthcare services if at all?**

- How might they impact on your role within the healthcare service?
- What impact might predictive tests have on current healthcare resources?

1. **How do you think healthcare services could be set up to provide predictive approaches for RA most effectively?**
   - What resources might be most beneficial to allocate to existing healthcare services to facilitate integration of predictive approaches?
2. **In what situations would an increased ability to predict RA be most useful?**
   - Why?
3. **What is most important to predict: Development of RA? Time to onset of RA? Severity of RA? Other outcomes?**
4. **What issues or concerns would you have about doing tests to predict future development of RA in people who don’t currently have RA?**

- What issues are there with integrating it into clinical practice?

1. **What benefit might there be in predicting future development of RA in people who don’t currently have RA?**

- What benefits might there be to integrating this into clinical practice?

1. **What type of healthcare professional do you think would provide predictive tests?**
   - What are your reasons for thinking this?

**Preventive Treatment**

**General introduction:**

- What do you currently know about interventions to prevent the development of RA?

Preventive interventions refer to any form of intervention that can lower the likelihood of developing a specific disease. These interventions can take the form of lifestyle interventions to decrease disease risk through, for example, changing nutrition and physical activity, or smoking cessation. Alternatively, drug treatments can be provided to lower RA risk. There are currently trials exploring the preventive efficacy of treatments such as hydroxychloroquine, methotrexate, rituximab and abatacept for those who are at different stages of risk for developing RA.

The following questions will relate to both lifestyle-interventions and pharmacological treatments, unless specifically stated otherwise. If your opinion differs for each type of intervention, feel free to mention this and explain why.

**Questions:**

1. **What are your views regarding the potential for preventive interventions for RA? Do you think RA can be prevented in people at risk?**
2. **What type of healthcare professional do you think would provide preventive interventions?**
   - What are your reasons for thinking this?
   - Prompt – is there a difference in your answer between drug treatments and another type of intervention?
3. **In what situation would you be most likely to suggest a lifestyle mediated preventive intervention such smoking cessation?**

- Why?

1. **In what situation would you be most likely to suggest a preventive treatment in the form of a pharmacological agent such as methotrexate?**
   - Why?
2. **How do you think the introduction of preventive interventions would affect healthcare services if at all?**

- How might they impact on your role within the healthcare service?
- What impact might preventive treatment have on current healthcare resources?

1. **How do you think healthcare services could be set up to provide preventive approaches for RA most effectively?**
   - What resources might be most beneficial to allocate to existing healthcare services to facilitate integration of preventive approaches?
2. **What issues might there be surrounding the introduction of these interventions into clinical practice?**
   - Prompt: concerns about risks of interventions especially re: drug treatments. What’s the acceptable risk of the treatment or of the development of RA for it to be used?
   - Issues surrounding introduction for those at risk of developing RA?
3. **What benefits might there be surrounding the introduction of these interventions to clinical practice?**
   - Benefits surrounding introduction for those at risk of developing RA?
4. **What level of risk for developing RA should a patient have for a preventive intervention to be considered?**
   - E.g. high/ low/intermediate risk?
   - What are your reasons for thinking this?
5. **How would a patient’s risk status affect the type of preventive intervention you would prescribe?**
   - E.g. would lower risk make you more likely to prescribe lifestyle, or drugs?
   - 20%, 50%, 70% risk?
6. **What level of benefit should a preventive intervention offer to be considered?**
   - E.g. complete prevention, delayed onset?
7. **How long should preventive interventions be recommended for?**
   - What are your reasons for thinking this?
   - Prompt: Is there a difference in your answer between drug treatments and another type of intervention?
